# Integrated transcriptome and metabolome profiling of *Camellia reticulata* reveal mechanisms of flower color differentiation

**DOI:** 10.3389/fgene.2022.1059717

**Published:** 2022-11-22

**Authors:** Fang Geng, Ruimin Nie, Nan Yang, Lei Cai, YunChong Hu, Shengtong Chen, Xiaomao Cheng, Zhonglang Wang, Longqing Chen

**Affiliations:** ^1^ College of Landscape Architecture and Horticulture Sciences, Southwest Landscape Architecture Engineering Technology Research Center of National Forestry and Grassland Administration, Yunnan Functional Flower Resources and Industrialization Technology Engineering Research Center, Southwest Forestry University, Kunming, Yunnan, China; ^2^ Kunming Institute of Botany, Chinese Academy of Science, Kunming, Yunnan, China

**Keywords:** omics, anthocyanins, carotenoids, flower, *Camellia reticulata*

## Abstract

*Camellia reticulata* (Lindl.) is an important ornamental plant in China. Long-term natural or artificial selections have resulted in diverse phenotypes, especially for flower colors. Modulating flower colors can enhance the visual appeal and economic value in ornamental plants. In this study, we investigated the molecular mechanisms underlying flower color differentiation in *C. reticulata*. We performed a combined transcriptome and metabolome analysis of the petals of a popular variety *C. reticulata* (HHYC) (red), and its two cultivars “Xuejiao” (XJ) (pink) and “Tongzimian” (TZM) (white). Targeted metabolome profiling identified 310 flavonoid compounds of which 18 anthocyanins were differentially accumulated among the three samples with an accumulation pattern of HHYC > XJ > TZM. Likewise, transcriptome analysis showed that carotenoid and anthocyanin biosynthetic structural genes were mostly expressed in order of HHYC > XJ > TZM. Two genes (*gene-LOC114287745765* and *gene-LOC114289234*) encoding for anthocyanidin 3-O-glucosyltransferase are predicted to be responsible for red coloration in HHYC and XJ. We also detected 42 MYB and 29 bHLH transcription factors as key regulators of anthocyanin-structural genes. Overall, this work showed that flavonoids, particularly anthocyanins contents are the major determinants of flower color differentiation among the 3 *C. reticulata* samples. In addition, the main regulatory and structural genes modulating anthocyanin contents in *C. reticulata* have been unveiled. Our results will help in the development of *Camellia* varieties with specific flower color and quality.

## 1 Introduction

*Camellia reticulata* (Lindl.), also known as Yunnan camellia is among the decorative trees belonging to the Camellia genus, which are members of the Theaceae family (Xia, 2003; Xin et al., 2015). There are 97 different species of Camellia, 76 of which are endemic to China (Yu, 1985; Xia, 2003; Zhang et al., 2019). China is considered the origin and distribution hub of *Camellia*. Generally, members of the genus *Camellia* are classified into one of five groups. Yunnan camellia (*C. reticulata*) and its near relatives constitute a significant group that is primarily found in Yunnan Province. In Yunnan province, *Camellia* is regarded as an exceptional ornamental plant with high economic values. Some of them are very important flowering ornamentals that also produce oil, and have a wide variety of cultivars (Silan et al., 2013; Xin et al., 2015). In addition to its cultural and medicinal significance, *C. reticulata* is also a valuable source of food products (Xia, 2003; Silan et al., 2013; Xin et al., 2015). *C. reticulata* is mostly red-colored, and there are few accounts of color variations in the species. As a result, producers and researchers have constantly sought to breed *C. reticulata* of diverse colors to enhance its aesthetic value (Xin et al., 2015).

Flower color is a key feature of horticultural crops, directly linked to pigments’ content and distribution (Zhao and Tao, 2015). Numerous studies have revealed that carotenoids, flavonoids, and alkaloids are among the most important pigments that contribute to the development of flower colors (Wang et al., 2001; Ma et al., 2018; Zhu et al., 2019). Particularly, carotenoids and flavonoids have garnered increasing interest as they are pigments extensively dispersed in plants and are responsible for the spectacular hues found in petals (Mekapogu et al., 2020; Cui et al., 2021; Gao et al., 2021; Mei et al., 2021). Carotenoids can cause flowers to display a wide spectrum of colors, from vivid red to orange to yellow (Clotault et al., 2008; Tanaka et al., 2008; Zhao and Tao, 2015). Likewise, flavonoids, which are the most significant secondary metabolites in plants, can create colors ranging from light yellow to purple, depending on the type of flavonoids. *Camellia* blooms have been the subject of recent studies due to the plant’s status as an ornamental plant. Carotenoids and flavonol glycosides were revealed to be the primary pigments in the golden yellow blooms of the *C. nitidissima* by Zhou et al. (2017). The pigment composition of red-flowered *Camellia* species was investigated by Jianbin Li et al. (2013), who concluded that cyanidin-core structure pigments (such as cyanidin 3,5-di-O-glucoside) have the potential to produce the most dominant phenotype among wild red-flowered *Camellia* species in China. According to Norihiko et al. (2001), the red blossoms of *Benibana-cha* also contain anthocyanins, albeit in somewhat different proportions. In spite of findings from a recent study that anthocyanins are not present in many white and yellow flowers of ornamental plants (Chen et al., 2012), practically all of the core metabolites needed for anthocyanin production are found in the white grape hyacinth petal (Lou et al., 2014). In a similar vein, the sepals of developing white tea flowers (*C. sinensis*) contain maximum levels of proanthocyanidins between the stages of complete separation and full bloom (Joshi and Gulati, 2011; Yu et al., 2020).

Anthocyanins are one of the most significant secondary metabolites that plants produce, and research has shown that they possess anti-cancer, anti-oxidation, and anti-atherosclerosis characteristics (Escribano-Bailon and Santos-Buelga, 2012; Jiang et al., 2021). Anthocyanins are responsible for the orange, red, magenta, violet, and blue flower colorations. The biochemical route that leads to petal pigment accumulation has been thoroughly studied, and the genes that code for the necessary enzymes and transcriptional factors have been identified (Escribano-Bailon and Santos-Buelga, 2012; Feng et al., 2013). Extensive research on the molecular mechanisms involved in the anthocyanin biosynthesis pathway (ABP) has been conducted. However, the majority of previous studies focused mostly on the coloration of fruits and leaves, while anthocyanin production in colored-petals is yet to be studied in *C. reticulata*. Anthocyanin biosynthesis and accumulation is a highly complicated process that is controlled by a number of enzymes, transcription factors, and influenced by varied external environmental factors such as light, water stress, and temperature (He and Giusti, 2010; Hu et al., 2015; Gao et al., 2021). As a result, additional research is required to get complete insights into the mechanism that underlies petal coloration in *C. reticulata*.

Early biosynthetic genes (EBGs) and late biosynthetic genes (LBGs) are the two categories of ABP genes that are found in dicots, along with *phenylalanine ammonia-lyase (PAL)* genes (Medda et al., 2021). The genes, *chalcone synthase (CHS), chalcone isomerase (CHI)*, and *flavanone 3-hydroxylase (F3H)* that are part of the common flavonoid biosynthesis pathway are included in the EBGs. These genes have an effect on downstream flavonoids; while LBGs consist of *F3′H, flavonoid 3′, 5′ hydroxylase (F3′5′H), dihydroflavonol 4-reductase (DFR)*, and *anthocyanidin synthase (ANS)* as well as *UDP-glucose:flavonoid 3-O-glucosyltransferase (UFGTs)* (Ren et al., 2021). Flowers come in a variety of colors, and these colors are determined by a number of factors, including co-pigments, cell structure, pH, and other factors (Stavenga et al., 2021). There is a correlation between the *MYB-bHLH-WD40* (MBW) complex and the transcriptional regulation of ABP. MYB (Allan et al., 2008; Fraser et al., 2013; Naik et al., 2021) and bHLH (Li et al., 2020a) are two of the components of this complex that act as positive regulators of ABP genes. In most cases, the expressions of these two components are unique to pigmented tissues. On the other hand, WD40 is found to perform analogous functions in pigmented and non-pigmented tissues alike. Despite this, WD40 is able to maintain the stability of the MBW complex (Hichri et al., 2011; Li et al., 2020b).

Transcriptome and metabolome high-throughput sequencing technology have been widely used in recent years to explore diverse plants’ color mechanisms. We performed transcriptome and metabolome profiling, annotation, and bioinformatics analysis to understand the molecular mechanism of *C. reticulata* petal color formation and differentiation. The results of this study will aid in the breeding of cultivars with specific-flower colors.

## 2 Materials and methods

### 2.1 Plant materials

Materials tested were *C. reticulata* flowers harvested from three different genotypes that had distinct blossom colors: A popular *C. reticulata* stock (red flower, ‘HHYC’) and its two cultivars *C. reticulata* ‘Xuejiao’ (pink flower, XJ), and *C. reticulata* ‘Tongzimian’ (white flower, TZM). Plant materials were collected in Zixi Mountain of Chuxiong (H: 2133m, E: 101°24′1″, N: 25°0′7″), Yunnan, China. They were planted at the experimental station of the College of Landscape and Horticulture, Southwest Forestry University (H:1950m, E:102°45′53″, N:25°4′0″), Kunming, Yunnan, China. Flowers were sampled during blooming in March 2021. There was a total of 9 *C. reticulata* flowers (petaLs) used in this experiment. As a result, all of the data were acquired based on the three distinct biological replicates that were gathered for each sample. All of the fresh *C. reticulata* petals were flash frozen in liquid nitrogen, and then placed in an ice chest and stored in −80°C refrigerator until further use.

### 2.2 RNA sequencing

The methods for isolating RNA, purifying, and monitoring it, as well as building a cDNA library and sequencing, were adapted from Sterling et al. (2015). The Qubit^®^ RNA Assay Kit in Qubit^®^ 2.0 Flurometer (Life Technologies, Carlsbad, CA, United States), the RNA Nano 6000 Assay Kit of the Agilent Bioanalyzer 2100 system (Agilent Technologies, Santa Clara, CA, United States), and the NanoPhotometer^®^ spectrophotometer (IMPLEN, Westlake Village, CA, United States) were used to check, measure, and evaluate the purity, concentration, and integrity. The sequencing libraries were prepared with the NEBNext^®^ UltraTM RNA Library Prep Kit for Illumina^®^ (NEB, Ipswich, MA, United States) in accordance with the recommendations by the manufacturer, and they were sequenced using an Illumina Hiseq platform. The NEBNext^®^ UltraTMRNA Library Prep Kit for Illumina^®^ (NEB, Ipswich, MA, United States) was used to prepare sequencing libraries, which were then sequenced using an Illumina Hiseq platform. This was done in accordance with the instructions provided by the manufacturer.

### 2.3 Identification of differentially expressed genes and transcription factors

With the help of the iTAK software (Zheng et al., 2016), transcription factors were identified. Previous methods by Zhao et al. (2012); Fraser et al. (2013) and Li et al. (2018) were adapted for both the identification and classification of transcription factors. Using a model of negative binomial distribution as a foundation, the *DESeq* R package (1.10.1) (Anders, 2010) was utilized to perform differential expression analysis. The false discovery rate was purposefully kept under control by the altered *p* values, which were changed using the methodology developed by Benjamini and Hochberg (1995). Genes were considered to have differential expression if their adjusted *p*-value was less than 0.05.

### 2.4 Real-time quantitative PCR

The RevertAidTM First Strand cDNA synthesis kit was utilized to analyze approximately 1 μg of total RNA in preparation for cDNA synthesis (Thermo Fisher Scientific, Waltham, MA, United States). An amplified reaction was carried out in a volume of L using the LightCycler^®^ 480 real-time PCR equipment in conjunction with a 96-well plate. Each reaction mix contained 1.0 μl of cDNAs, SYBR Premix ExTaq™ 10 μl, PCR forward primer (10 μmol·L- 1) 0.5 μl, PCR reverse primer (10 μmol·L^− 1^) 0.5 μl, ddH_2_O 8.0 μL, to a final volume of 20 μL. The amplified reaction began with 95°C, maintained for 5 min, and was then followed by 45 cycles of 10 s at 95°C, 20 s at 60°C, and 20 s at 72°C. After each experiment was finished, a melt-curve analysis was performed with the default parameters (5 s at 95°C and 1 min at 65°C). Normalization was accomplished with *β-actin* gene as control (Selvey et al., 2001; Tang et al., 2022). Every analysis was carried out in triplicate with separate biological replicates serving as controls. Table 1contains a listing of the primer sequences.

**TABLE 1:** Primer sequences used for the qRT-PCR validation.

Gene name	Forward primer	Reverse primer
gene-LOC114289234	TCA​ATC​ACT​TCC​ATT​CCT​CTC​A	CGA​GTG​CTA​ACT​CCC​ATT​TCT​T
gene-LOC114285765	CAG​TTG​ATT​CCC​CAG​TGA​TAC​A	TTT​ATC​TAG​GCA​TGA​GGG​TGC​T
gene-LOC114285774	GGT​ATT​TCC​ATC​TCA​GGA​TTG​C	TAC​CTT​ACC​TAG​ACC​TGG​CGA​A
gene-LOC114288492	TTC​CTT​TTC​ATG​GTT​GTA​GAG​C	GAA​CTG​GAT​CGT​GTA​ACT​TCA​TTC
gene-LOC114277682	GTA​CCC​GTC​GTA​TTT​GCT​TCT​C	GAT​CAT​ATT​TGT​GCA​GTG​TCG​G
gene-LOC114287489	GTA​TCC​AAA​TCC​ATG​CAA​CTC​A	TTC​CCC​ATA​GCC​AAG​AAT​AGA​A
gene-LOC114299010	CGT​ATC​TGT​GGC​TAC​TGC​TGT​T	ATC​TCC​CGC​TCC​AGG​TAT​TAT​T
gene-LOC114300537	TTG​CCC​CAT​AAT​ACA​GAA​CCT​C	AGG​AGA​AAA​GGA​GGC​ATT​TAC​C
gene-LOC114316923	TGG​CGA​GAT​ATA​CCC​CAT​CTA​T	ACT​ACG​CCT​ATT​GCT​TAG​ACC​G
gene-LOC114267429	TCA​ATC​ACT​TCC​ATT​CCT​CTC​A	CGA​GTG​CTA​ACT​CCC​ATT​TCT​T
gene-LOC114322364	CGA​GAA​GGG​CTT​ATT​TGA​CCT​A	GAA​AGG​AAG​AAC​TCA​CGC​TTT​T
gene-LOC114280872	TCT​ATC​ACT​TTG​CCC​CTC​TTT​T	TTC​GTT​GAG​AAA​ACA​ACC​TAC​G
gene-LOC114268760	AGC​CAC​TAT​GAG​CCT​ACT​GCA​T	GAA​CCG​ATG​ACT​TAC​GCC​TTA​C
gene-LOC114257730	CAA​CAC​CAC​CAA​CGC​ATA​TC	AGC​AAC​GAT​TCA​CGA​CAT​TG
gene-LOC114257731	GGG​GTG​AGT​ATT​TCG​GGA​GT	TCC​ATG​CAT​CTT​TTA​CAC​TGA​A
gene-LOC114257999	GTC​CGT​CAA​CTC​GAT​CAC​CT	TTC​AAC​CAA​ACC​CCA​TCA​TT
gene-LOC114258462	GCC​ATT​TCT​TCA​TTT​GGT​GC	GCA​TTT​CAA​TTT​TTC​ACC​CC
gene-LOC114258463	TGG​ACA​AAG​ACA​CAA​TCA​CAC​A	TTG​AAT​TTC​GAT​CTT​TCC​ATC​A
β-actin	CTG​ATG​CAA​AAA​CTG​CCA​AA	ACC​GCA​CTC​AAA​GGT​TCA​AT

### 2.5 Extraction of metabolites

Metabolites were extracted using a 50% methanol buffer after the samples were thawed on ice (50% solution of methanol in distilled water). Following incubation at room temperature for 10 min and vortexing for 1 min to extract the 20 μl of sample in 120 μl of precooled 50% methanol, the extraction liquid was stored overnight at 60°C. In 96-well plates, the supernatants were transferred following a 20-min centrifugation at 4000 rpm. The extraction and subsequent analysis of data was conducted in three separate instances. An ultra-performance liquid chromatography (UPLC) system was used for all chromatographic separations (SCIEX, Cheshire, United Kingdom). The reversed phase separation was performed using an ACQUITY UPLC BEH Amide column (100 mm × 2.1 mm, 1.7 m, Waters, United Kingdom). The temperature of the column oven was kept at 35°C. Solution A (25 mM ammonium acetate +25 mM NH_4_H_2_O) and Solution B were used as the mobile phase. The flow rate was 0.4 ml/min. The following were the gradient elution conditions: 95% B for the first 0.5 min; 95%–65% B for the next 0.5–9.5 min; 65%–40% B for the next 9.5–10.5 min; 40% B for the next 10.5–12 min; 40% B for the next 12–12.2 min; and 95% B for the next 12.2–15 min. Each sample received a 4 L injection. The metabolites eluted from the column were detected using a high-resolution tandem mass spectrometer TripleTOF5600plus (SCIEX, Cheshire, United Kingdom). Positive and negative ion modes of the Q-TOF were both utilized. The chromatograph and mass spectrometer were controlled by XCMS software 3.2.0 (UC, Berkeley, CA, United States).

### 2.6 Detection and evaluation of metabolite concentrations

The MSConvert software was utilized to convert raw LC-MS data into the mzXML format, which was further processed by the XCMS, CAMERA, and metaX toolboxes, which were all integrated within the R program (Li et al., 2018; Stanstrup et al., 2019). To identify each ion, the combined retention time (RT) and m/z data was utilized. The Plant Metabolic Pathway Databases (PLANTCYC; http://www.plantcyc.org/), Kyoto Encyclopedia of Genes and Genomes (KEGG; http://www.kegg.jp/), and Saskatoon Public Library in-House Databases (http://spldatabase.saskatoonlibrary.ca/), and Human Metabolome Databases (HMDB; http://www.hmdb.ca/) were used to perform level-one and level-two identification and annotation to explain the physico-chemical properties as well as the biological functions of metabolites. The metaX software (http://metax.genomics.cn/), was employed for screening and quantitative analysis of differential metabolites.

### 2.7 Statistical analysis

The relative gene expressions were computed utilizing the 2^−ΔΔCT^ method (Livak and Schmittgen, 2001), and data visualization was performed utilizing GraphPad Prism 5 (GraphPad Software Inc., San Diego, California, United States). Both the heatmap and the cluster analyses were performed with the help of R-3.4.2 and MEGA6, respectively. For the analysis of significant differences, IBM SPSS (IBM SPSS Software Inc., San Diego, California, United States) was used.

## 3 Results

### 3.1 Flower color variation among *Camellia genotypes*

**FIGURE 1:**
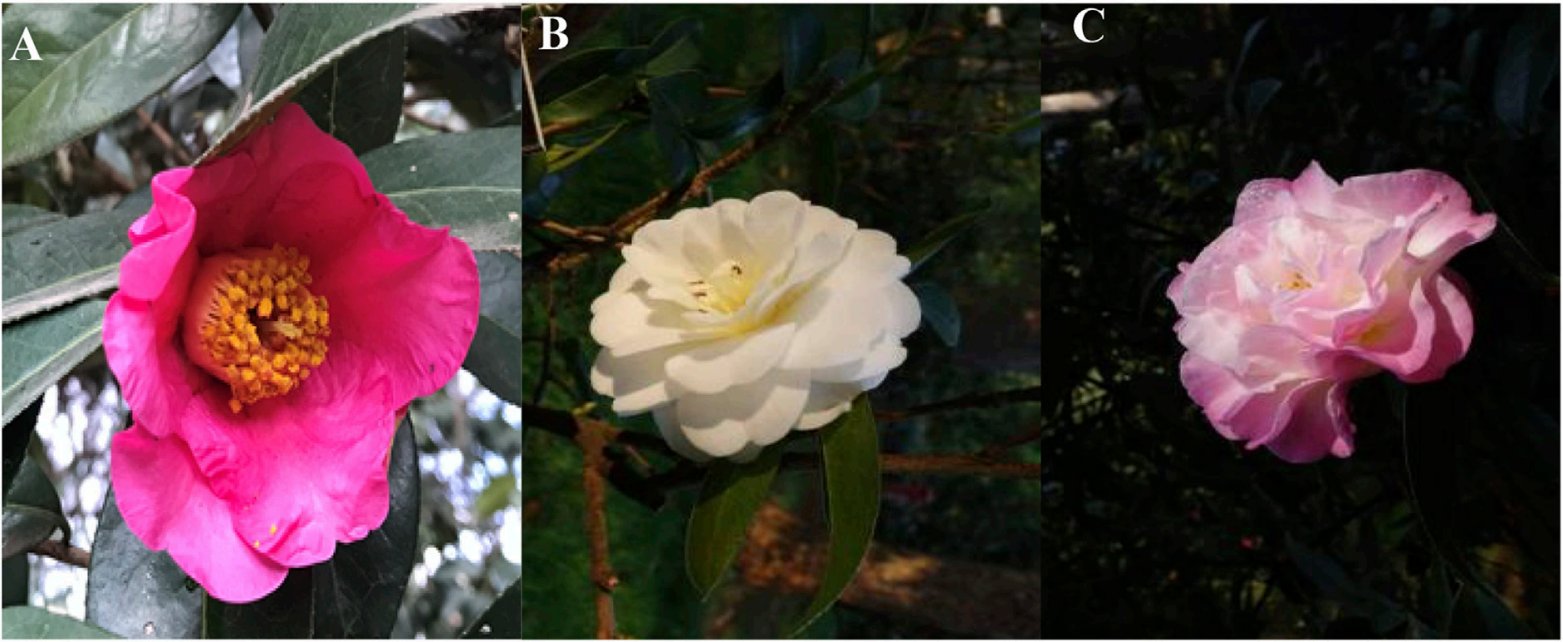
Different flower colors among three *Camellia reticulata.* **(A)** *C. reticulata* (HHYC), **(B)** *C. reticulata* ‘Tongzimian’ (TZM), **(C)** *C. reticulata* ‘Xuejiao’ (XJ).

In attempt to understand the molecular mechanism underlying the color formation and differentiation in *C. reticulata*, we performed transcriptome and metabolome profiling of petals from *C. reticulata* (HHYC), *C. reticulata* ‘Xuejiao’ (XJ) and *C. reticulata* ‘Tongzimian’ (TZM). The petals of HHYC and TZM are red and white, respectively (Figures 1A,B), while the petals of XJ are the intermediate of HHYC and TZM, thus pink (red-white) color (Figure 1C).

### 3.2 Transcriptome profiling of petals from three contrasting flowers of *C. reticulata*

The transcriptome profiling was performed on nine libraries (3 genotypes) with contrasting petals/flowers × 3 biological repeats) leading to an average of 47, 49 and 48 Gb data for HHYC, XJ and TZM, respectively (Table 2). Of these, an average of 45,500,491 bp were clean reads with an error rate of 0.02% (Table 2). Furthermore, the Q30 (%) and guanine-cytosine content ranged from 94.55 to 95.15 and 44.51–45.30%, respectively (Table 2). These results suggest that the transcriptome results qualified for further downstream analyses.

**TABLE 2:** Summary of RNA-Seq data and mapping metrics of three contrasting flowers of *C. reticulata*.

Sample^a^	Raw reads (bp)	Clean reads (bp)	Clean base (Gb)	Error rate (%)	Reads mapped (%)	Unique mapped (%)	Q30 (%)^b^	GC (%)^c^
HHYC-1	48998284	47530980	7.13	0.02	75.54	71.12	94.90	45.30
HHYC-2	45727170	44108884	6.62	0.02	74.17	69.8	94.70	45.27
HHYC-3	48079296	42457662	6.37	0.02	73.58	69.03	94.81	45.28
HHYC	47601583	44699175	6.71	0.02	74.43	69.98	94.80	45.28
XJ-1	46926224	42171752	6.33	0.02	73.26	68.78	95.15	44.88
XJ-2	54057590	51492124	7.72	0.02	74.02	69.34	94.55	44.68
XJ-3	46145294	44832014	6.72	0.02	73.46	68.45	94.91	44.65
XJ	49043036	46165297	6.92	0.02	73.58	68.86	94.87	44.74
TZM-1	47604800	45981370	6.9	0.02	74.5	69.62	94.90	44.51
TZM-2	45923820	43484386	6.52	0.02	73.89	69.28	95.15	44.76
TZM-3	51788184	47445248	7.12	0.02	73.93	69.32	95.06	44.81
TZM	48438935	45637001	6.85	0.02	74.11	69.41	95.04	44.69
Average	48361185	45500491	6.83	0.02	74.04	69.42	94.90	44.90

^a^ Sequencing was done in triplicate of *C. reticulata* (HHYC1-3), *C. reticulata* ‘Xuejiao’ (XJ1-3) and *C. reticulata* ‘Tongzimian’ (TZM1-3), with HHYC, XJ, and TZM, representing the means.

^b^ Quality control at ≤ 30%.

^c^ Guanine and cytosine content.

**FIGURE 2:**
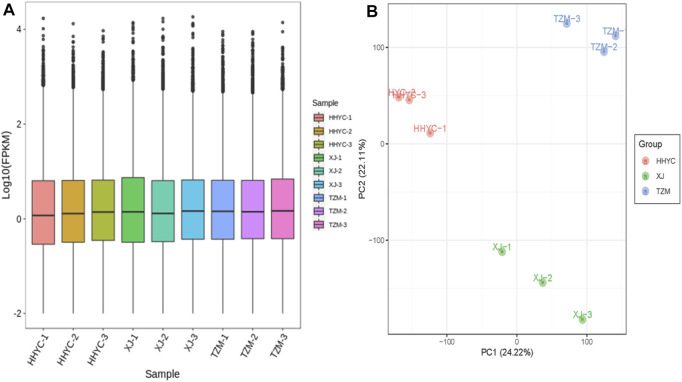
Quantification of fragments per kilobase of exon per million fragments mapped (FPKM) among the nine samples (3 contrasting flowers × 3 biological replicates). **(A)**. FPKM distribution among the nine samples. **(B)**. Principal components analysis of three contrasting flowers of *C. reticulata* (HHYC1-3), *C. reticulata* ‘Xuejiao’ (XJ1-3) and *C. reticulata* ‘Tongzimian’ (TZM1-3).

In all, 61,277 genes comprising 44,751 known genes and 16,526 novel genes were detected from the nine libraries (Supplementary Table S1). From these, 53,861, 53,739 and 54,631 genes were expressed in HHYC, XJ and TZM, respectively. The fragments per kilobase of exon per million fragments mapped (FPKM) distribution and principal component analysis (PCA) of the expressed genes are shown in Figures 2A,B. The Pearson correlation coefficients (r ≥ 0.81) and PCA based on FPKM showed high levels of reproducibility of petal samples from the same genotype (Supplementary Figure S1). Most of the expressed genes were successfully annotated to at least one of the nine public databases, i.e., Kyoto Encyclopedia of Genes and Genomes (KEGG), KEGG pathway, NCBI non-redundant (NR), Swiss protein (Swissprot), KEGG orthologous (KOG), Gene ontology (GO), Protein family (Pfam) and Transcription factor (TF) family (Supplementary Table S1). The successful annotation of most of the identified genes coupled with high reproducibility and repeatability of petal samples from the same genotype give credence to our transcriptome results for the identification of differentially expressed genes (DEGs) and subsequent analyses.

### 3.4 Differentially expressed genes and kyoto encyclopedia of genes and genomes pathway analyses

**FIGURE 3:**
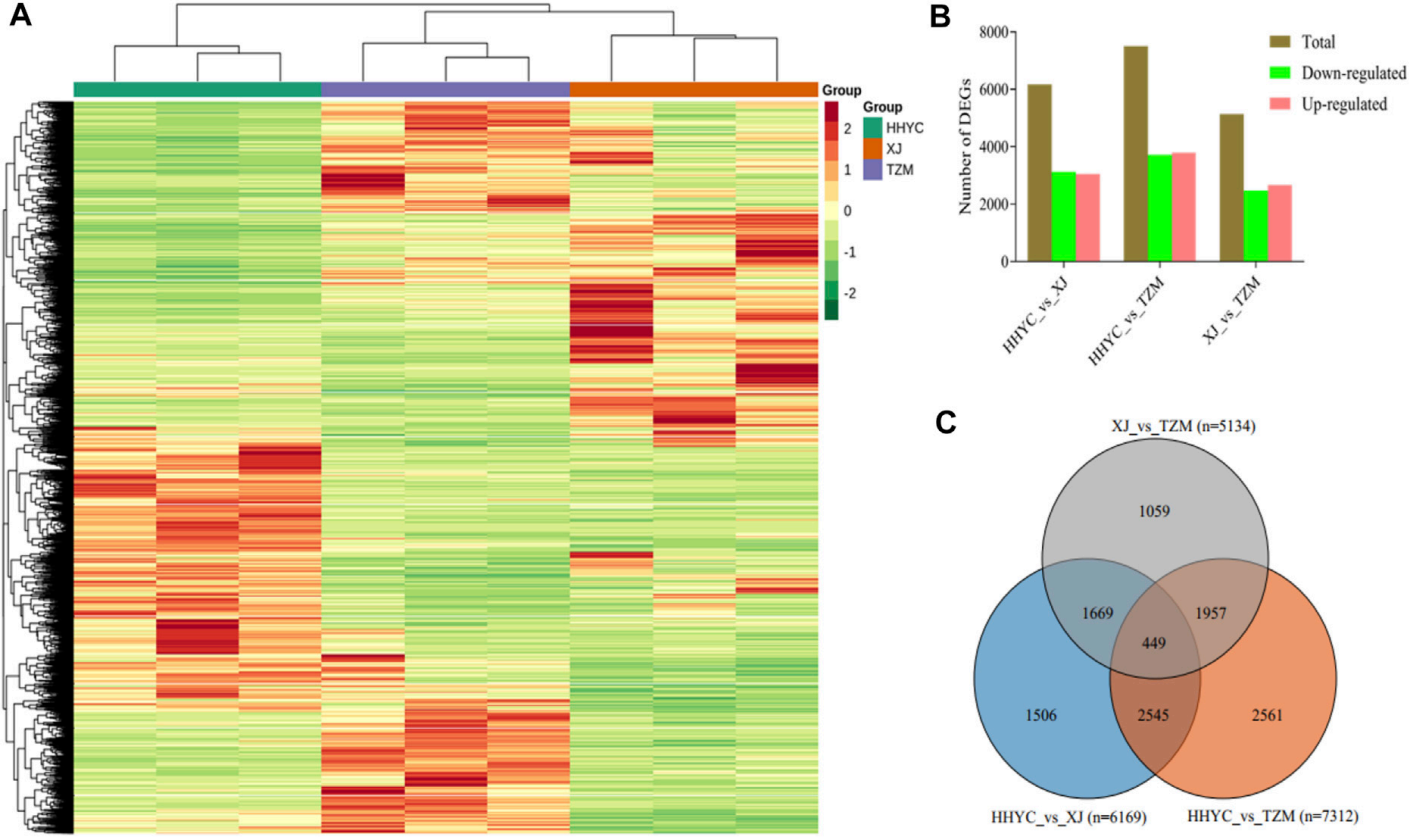
Differentially expressed genes in the three contrasting flowers of *C. reticulata* (HHYC), *C. reticulata* ‘Xuejiao’ (XJ) and *C. reticulata* ‘Tongzimian’ (TZM). **(A).** Hierarchical clustering heatmap based on fragments per kilobase of exon per million fragments mapped of differentially expressed genes (DEGs) of the three samples (HHYC, XJ and TZM) based on triplicate biological repeats. The red, blue and green colors on column cluster (shown right hand side) represent HHYC, XJ and TZM, respectively. **(B)**. Extent of regulation of DEGs. **(C)**. Venn diagram of DEGs among the three pairwise group comparisons (n = number of DEGs detected).

A total of 11,746 DEGs were detected among the HHYC, XJ and TZM genotypes in pairwise comparisons upon application of strict criteria of log2 fold change (log2FC) ≥ 1 and false discovery rate (FDR) with adjusted *p*-value (padj) < 0.05. We subsequently performed hierarchical clustering heatmap of biological replicates from the three genotypes based on the FPKM values of the DEGs, the nine samples were divided into two major groups with each biological repeat in the sub-group. The two major groups comprised HHYC in group I, while XJ and TZM formed the group II (Figure 3A). The grouping pattern suggests that XJ and TZM have some level of similarity in terms of flower coloration mechanism (Figure 1). Among the pairwise groups, 6169, 7512 and 5134 DEGs were detected in HHYC_vs_XJ, HHYC_vs_TZM and XJ_vs_TZM, respectively (Supplementary Tables S2A-C; Figure 3B) with nearly equal proportion of up-and down-regulated genes. The least number of DEGs in XJ_vs_TZM further pinpoints the similarity in flower coloration mechanism. We also compared the results of DEGs in each pairwise group and a total of 449 core conserved DEGs were detected among the three pairwise groups (Figure 3C). These results highlight that XJ and TZM have similar flower coloration mechanism than HHYC.

In order to verify the transcriptome results, 18 genes were randomly selected for qRT-PCR to establish the correlation between gene expression profiles from the RNA-seq and qRT-PCR. The relative expression levels of the selected genes were mostly consistent with that of RNA-seq with correlation score of 84% (Supplementary Figures S2A,B). This further confirms the reliability of transcript quantification from our RNA-seq.

### 3.5 Analysis of key transcription factor families

It is well established that MYB-bHLH-WD40 (MBW) complex is involved in modulating coloration in plants (Fraser et al., 2013; Naik et al., 2021). However, in this study, no gene with WD40 was detected. Hence, we mined for MYB and bHLH transcription factors (TF) families among the DEGs and their log2 transformed FPKM were heatmapped. A total of 276 and 174 genes with MYB and bHLH TFs were identified, respectively, from which 42 MYB and 29 bHLH were detected among the DEGs. We further studied the expression profiles of these genes (Supplementary Figure S3A,B). For instance, the expression of *gene-LOC114321352*, *gene-LOC114268549*, *gene-LOC114264400* and *gene-LOC114281024* followed in HHYC or TZM were greater than XJ (Supplementary Figure S3A). In similar vein with rearrangement of the three genotypes by the 29 DEG encoding bHLH, *gene-LOC114233324*, *gene-LOC114258501*, *gene-LOC114298318*, *gene-LOC114287545* and *gene-LOC114319413* expressed lower in XJ relatively to either HHYC or TZM (Supplementary Figure S3A). The identified MYB-bHLH genes could be useful as genomic resources to deepen our understanding of transcriptional regulation flower coloration in *C. reticulata*.

### 3.6 Analysis of carotenoid biosynthetic pathway genes among three contrasting *C. reticulata* genotypes

**FIGURE 4:**
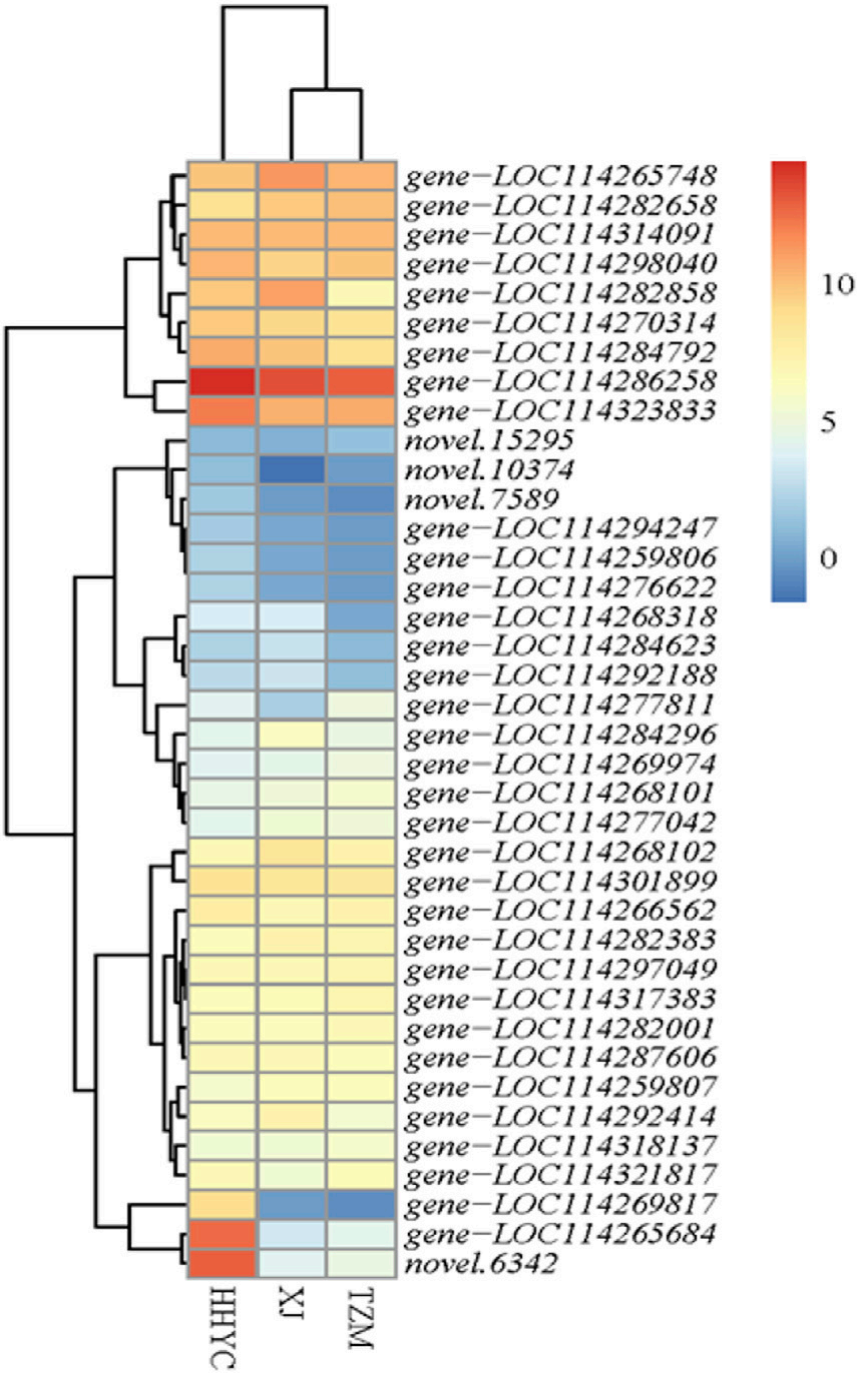
Heatmap clustering of thirty-eight structural genes involved in carotenoid biosynthesis among the three contrasting flowers of *C. reticulata* (HHYC), *C. reticulata* ‘Xuejiao’ (XJ) and *C. reticulata* ‘Tongzimian’ (TZM). The expression levels of the genes were log2 transformed from averages of the three biological repeats from each genotype and the transformed data were used to draw the heatmap. Color diagram at the right side shows the intensity of the gene expression.

Thirty-eight carotenoid biosynthetic structural genes were differentially expressed among the three pairwise groups. Their expression profiles were subjected to hierarchical clustering heatmap. The expression profiles of the 38 genes grouped the 3 genotypes of *C. reticulata* into 2 groups (Figure 4), just as the hierarchical clustering heatmap of the 11,746 DEGs in Figure 3A. It is reported that carotenoid gives yellow-orange-red color to fruits/flowers in plants (Mortensen, 2006; Tanaka et al., 2008). Two ortholog genes (*gene-LOC114265684* and *novel.6342*) of *At4g38540* encoding for zeaxanthin epoxidase (ZEP) on the carotenoid biosynthetic pathway expressed more than 10-fold higher in HHYC than either XJ or TZM (Figure 4), suggesting these genes could be candidate genes for red pigmentation in HHYC. Several studies have demonstrated that ZEP is the major contributor to carotenoid composition (Gonzalez-Jorge et al., 2016; Lee et al., 2021). From the Figure 4, another gene worth elaborating is *gene-LOC114269817* encoding for abscisate beta-glucosyltransferase (ABA-UGT) [EC:2.4.1.263]. It was nearly not expressed in either XJ or TZM. This suggests that *gene-LOC114269817* may contribute to red coloration in HHYC as a recent study by Li et al. (2022) revealed that increase in ABA-UGT gene contributed to red coloration in strawberry fruit, but its reduction contributed to white coloration in an unripe fruit.

### 3.7 Analysis of phenylpropanoid/flavonoid/anthocyanin biosynthetic pathways genes among the three contrasting *C. reticulata* genotypes

Aside carotenoid biosynthesis, it is well known that flavonoids are major molecules involved in plant pigmentation specifically anthocyanins (Lai et al., 2014). Phenylpropanoids are precursors of flavonoid/flavone and flavonol which lead to anthocyanins biosynthesis and accumulation. Therefore, we explored DEGs in the pairwise groups based on the KEGG pathway enrichment analyses (Supplementary Table S3). Sixteen structural genes involved in flavone and flavonol biosynthesis (Ko0094) were differentially expressed between HHYC and XJ (Supplementary Table S2A). Out of these 16 structural genes, *gene-LOC114309343* (flavonoid 3′-monooxygenase [EC:1.14.14.82]) and other 2 genes (*gene-LOC114273964* and *gene-LOC114273955*) encoding flavonol-3-O-glucoside/galactoside glucosyltransferase and anthocyanidin 3-O-glucoside 2″-O-glucosyltransferase-like, were not expressed in XJ genotype, making them potential candidate genes for the white pigmentation in XJ.

Flavonoid biosynthesis (Ko00941) was enriched with 76 (45 down-regulated and 31 up-regulated) and 40 (25 down-regulated and 15 up-regulated) DEGs in HHYC_vs_TZM and XJ_vs_TZM, respectively (Supplementary Tables S2, 3). The higher number of down-regulated genes indicate that flavonoid biosynthetic genes were more activated in colored petals than the white petals (Figure 1). Additionally, anthocyanins biosynthesis was enriched with four DEGs (*gene-LOC114289234*, *gene-LOC114285765*, *gene-LOC114285774* and *gene-LOC114288492*) encoding anthocyanidin 3-O-glucosyltransferase [EC:2.4.1.115]. The DEGs were expressed higher (2.22–18.14 times) in HHYC than in TZM (Supplementary Table S2A). Likewise, these genes expressed higher in XJ genotype than in TZM genotype (Supplementary Table S2A). These suggest that the weak expression of anthocyanidin 3-O-glucosyltransferase encoded genes are likely responsible for the white flower coloration in TZM genotype. These genes are involved in the conversion of pelargonidin, cyanidin and delphinidin to pelargonidin 3-glucoside, cyanidin 3-glucoside and delphinidin 3-glucoside, respectively (Supplementary Figures S4A,B).

### 3.8 Flavonoid-based metabolome profiling of flowers of the three contrasting *C. reticulata*

**FIGURE 5:**
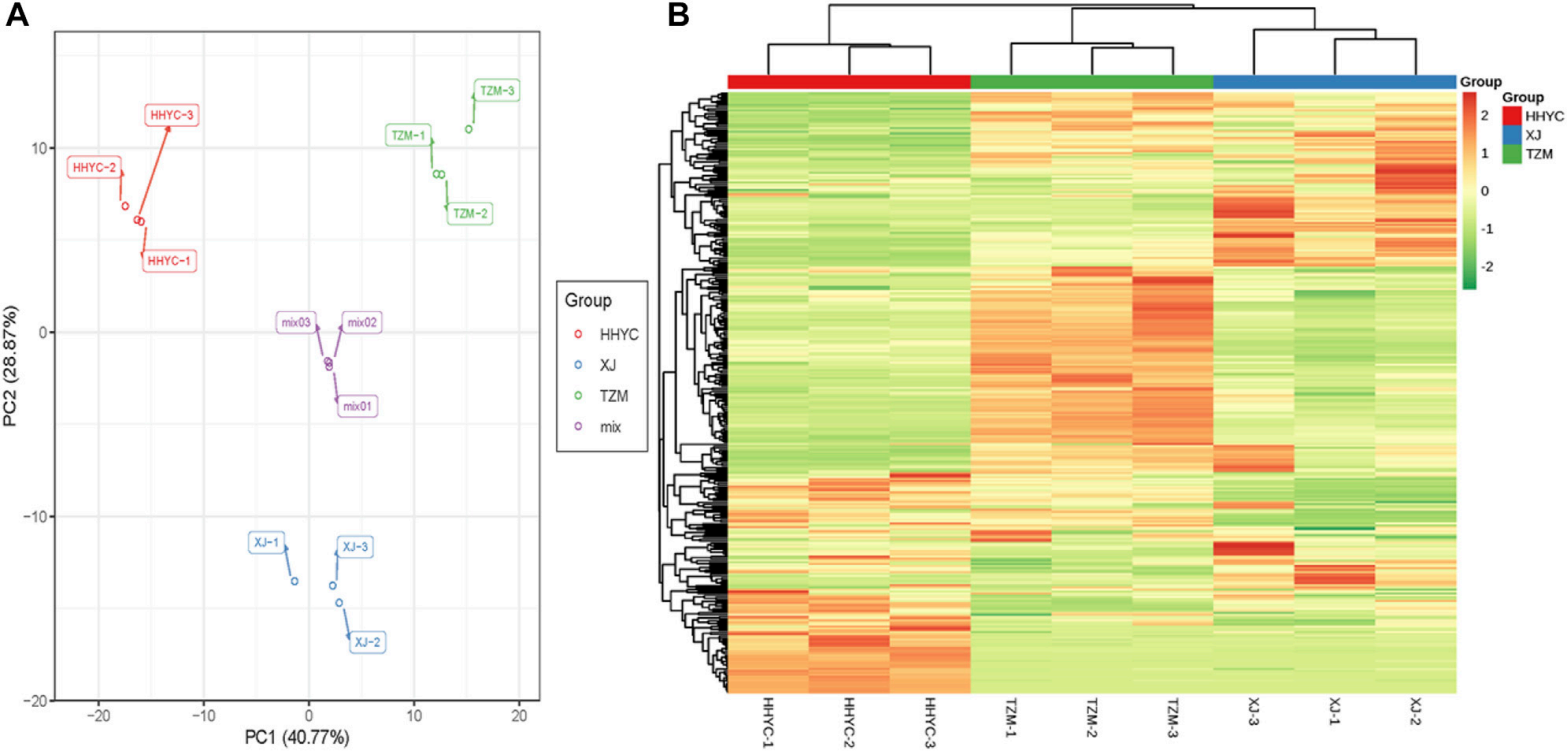
Profiles of metabolites accumulated among the 3 from petals of contrasting flowers of *C. reticulata* (HHYC; red colored), *C. reticulata* ‘Xuejiao’ (XJ; pink colored) and *C. reticulata* ‘Tongzimian’ (TZM; white colored). **(A)**. Principal component analysis based on metabolites normalized ion intensities. Mix samples represent equal proportion of samples from HHYC, XJ and TZM. Metabolome profiling was done in triplicates for each genotype (HHYC-1 to -3, XJ-1 to -3, TZM-1 to -3 and mix-1 to -3). **(B)**. Hierarchical clustering heatmap based on metabolites normalized ion intensities. The red, blue and green colors on column cluster (shown righthand side) represent HHYC, XJ and TZM, respectively. The ion intensity of each metabolite are shown green color represents lowly-accumulated, and red color represents highly-accumulated metabolites.

To further understand the metabolites involved in the reddish color formation in HHYC compared to the two other genotypes, we performed a targeted metabolome profiling of flavonoid compounds in the three genotypes. This led to a total of 310 metabolites (282 flavonoids and 28 tannins) detected (Supplementary Table S4). We performed PCA and hierarchical heatmap clustering based on the ion intensities of the 310 compounds. We observed that the grouping/topology (Figures 5A,B) followed the FKPM of genes obtained from the RNA-seq (Figure 3A).

To identify the differentially accumulated metabolites (DAMs), we performed partial least squares discriminant analysis (PLS-DA) with threshold of log2FC ≥1 and variable importance in projection (VIP) ≥1. From these stringent criteria, we detected 138, 149 and 133 DAMs in HHYC_vs_XJ, HHYC_vs_TZM and XJ_vs_TZM, respectively (Supplementary Figures S5A,B; Supplementary Table S5).

From above, 18 anthocyanin-based DAMs were detected with an accumulation pattern: HHYC > XJ > TZM (Table 3). All the 18 DAMs were detected in HHYC, while only nine and seven DAMs were detected in XJ and TZM genotypes, respectively. These trends suggest that anthocyanin is the major pigment responsible for the flower coloration among the three contrasting genotypes of *C. reticulata*. For instance, cyanidin 3-xyloside (Hmhp002834), cyanidin-3,5-O-diglucoside (cyanin) (pme1777), cyanidin-3-O-(6″-O-malonyl)glucoside (pmb0542), cyanidin-3-O-arabinoside (Smlp002532), cyanidin-3-O-galactoside (pmf0027), cyanidin-3-O-glucoside (kuromanin) (pmb0550), cyanidin-3-O-rutinoside (keracyanin) (pme1773), delphinidin-3,5-di-O-glucoside (pmf0116), delphinidin-3-O-arabinoside (Smlp001915), delphinidin-3-O-galactoside (Lmcp001821), delphinidin-3-O-glucoside (mirtillin) (pme1398), delphinidin-3-O-sophoroside-5-O-glucoside (Lmqp001553), pelargonidin-3-O-glucoside (pme3392), peonidin-3-O-(6″-O-p-coumaroyl)glucoside (Lmpp003929) and peonidin-3-O-glucoside (pmf0203) accumulated higher in HHYC genotype but were either completely absent or lower in either XJ or TZM (Table 3).

**TABLE 3:** Ion intensities of anthocyanin-based differentially accumulated metabolites among petals from three contrasting flowers of *C. reticulata*.

Index^a^	Compounds	HHYC^b^	XJ^c^	TZM^d^
Hmhp002834	Cyanidin 3-xyloside	27941333	24756	Absent
pme1777	Cyanidin-3,5-O-diglucoside (Cyanin)	30024333	36218	111577
Lmcp003751	Cyanidin-3-O-(6""-O-acetyl-2""-O-xylosyl) glucoside	6382	277067	Absent
Lmcp004347	Cyanidin-3-O-(6″-O-caffeoyl-2″-O-xylosyl) glucoside	38132	3406100	241843
pmb0542	Cyanidin-3-O-(6″-O-malonyl) glucoside	360783	Absent	Absent
Lmjp002491	Cyanidin-3-O-(6″-O-p-coumaroyl-2″-O-xylosyl) glucoside	771273	28742000	12310900
Smlp002532	Cyanidin-3-O-arabinoside	30060333	15886	Absent
pmf0027	Cyanidin-3-O-galactoside	13049667	28850	Absent
pmb0550	Cyanidin-3-O-glucoside (Kuromanin)	15752000	Absent	Absent
pme1773	Cyanidin-3-O-rutinoside (Keracyanin)	2957200	Absent	Absent
pmf0116	Delphinidin-3,5-di-O-glucoside	4171133	11328	37114
Smlp001915	Delphinidin-3-O-arabinoside	207653	Absent	Absent
Lmcp001821	Delphinidin-3-O-galactoside	18885000	Absent	158770
pme1398	Delphinidin-3-O-glucoside (Mirtillin)	18257667	Absent	145603
Lmqp001553	Delphinidin-3-O-sophoroside-5-O-glucoside	23826	Absent	Absent
pme3392	Pelargonidin-3-O-glucoside	22014000	Absent	Absent
Lmpp003929	Peonidin-3-O-(6″-O-p-coumaroyl) glucoside	1275933	29948	19105
pmf0203	Peonidin-3-O-glucoside	11068667	Absent	Absent
Average		10,936,962.05	3,619,128.11	1,860,701.81

^a^ Compound index from self-built database MWDB (Metware database).

^b^ *C. reticulata* (HHYC).

^c^ *C. reticulata* ‘Xuejiao’ (XJ). *C. reticulata* ‘Tongzimian’ (TZM). Ion intensities were computed as the average of the three biological repeats of each genotype.

On the contrary, cyanidin-3-O-(6″-O-caffeoyl-2″-O-xylosyl) glucoside (Lmcp004347) and cyanidin-3-O-(6″-O-p-coumaroyl-2″-O-xylosyl) glucoside (Lmjp002491) accumulated higher in XJ than in HHYC and TZM (Table 3), making candidate biomarkers to distinguish flowers of the three genotypes. The complete absence of cyanidin 3-xyloside (Hmhp002834), cyanidin-3-O-(6""-O-acetyl-2""-O-xylosyl) glucoside (Lmcp003751), cyanidin-3-O-arabinoside (Smlp002532) and cyanidin-3-O-galactoside (pmf0027) in TZM may be responsible for the flower color differentiation between XJ and TZM genotypes.

### 3.9 Conjoint analysis of transcriptome and metabolome results

**FIGURE 6:**
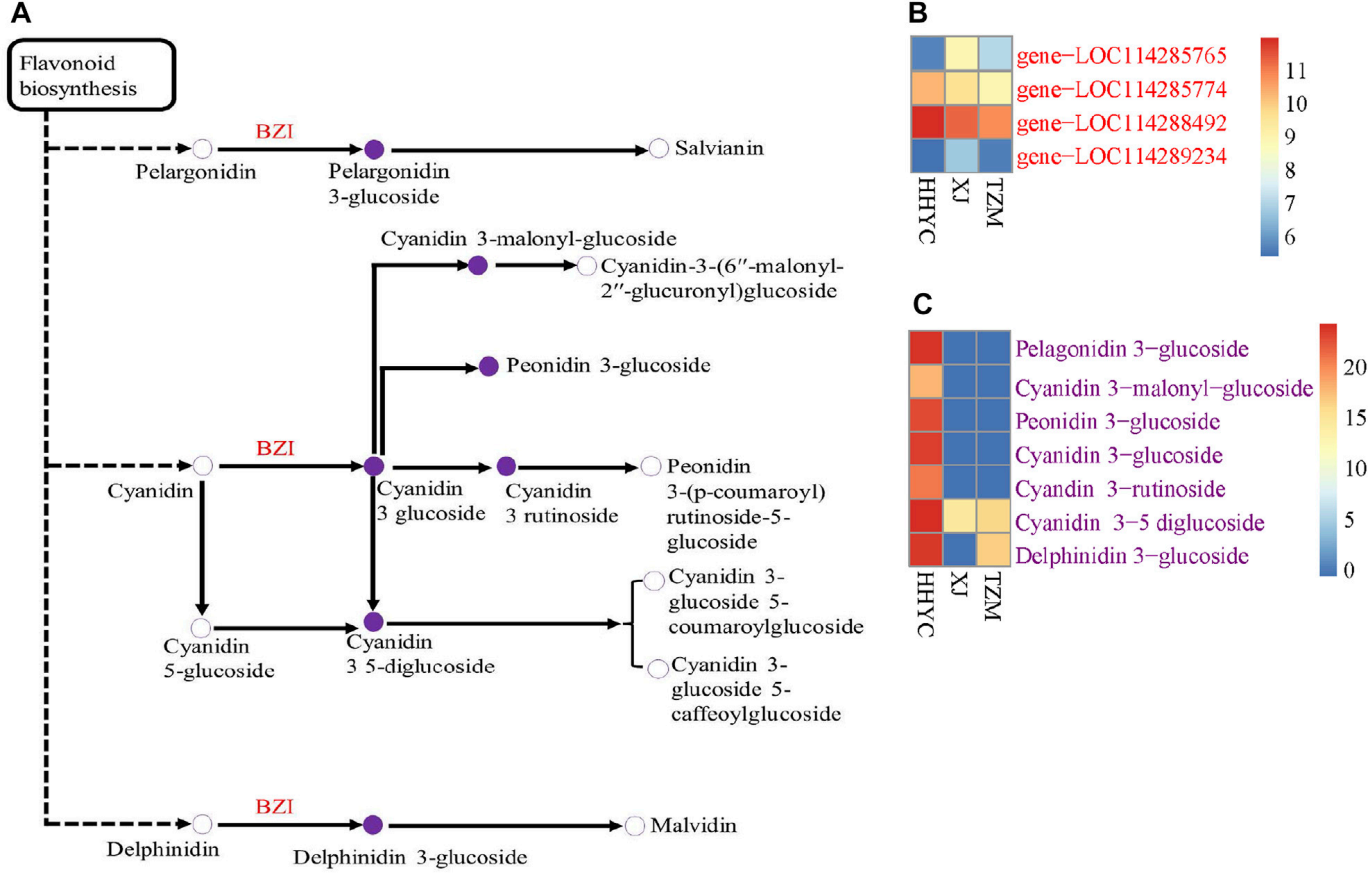
Schematic diagram of anthocyanins biosynthetic pathways responsible for color differentiation among *C. reticulata* (HHYC), *C. reticulata* ‘Xuejiao’ (XJ) and *C. reticulata* ‘Tongzimian’ (TZM). **(A)**. Heatmap of four anthocyanidin 3-O-glucosyltransferase [EC:2.4.1.115] (BZI) encoded genes. **(B)**. Heatmap of seven differentially accumulated metabolites. Metabolites with hollow circles were not detected in this study, while those in purple circles were detected in this study. Heatmaps were produced with log2 transformed data. Color diagram at the right side shows the intensity of the metabolite accumulation or gene expression.

We further performed a conjoint analysis of the transcriptome and metabolome data to assess the relationship between the genes and metabolites. This helps to statistically prioritize genes and metabolites that alter flower colors in *C. reticulata*. It was observed that anthocyanin is the key pigmentation that differentiates flowers of HHYC_vs_XJ and HHYC_vs_TZM (*p* = 0.004), however XJ_vs_TZM were not differentiated, possibly as a result of high similarity between the flowers of XJ and TZM as observed in the heatmap clustering (Figure 3A: Figure 5B; Figure 6).

**FIGURE 7:**
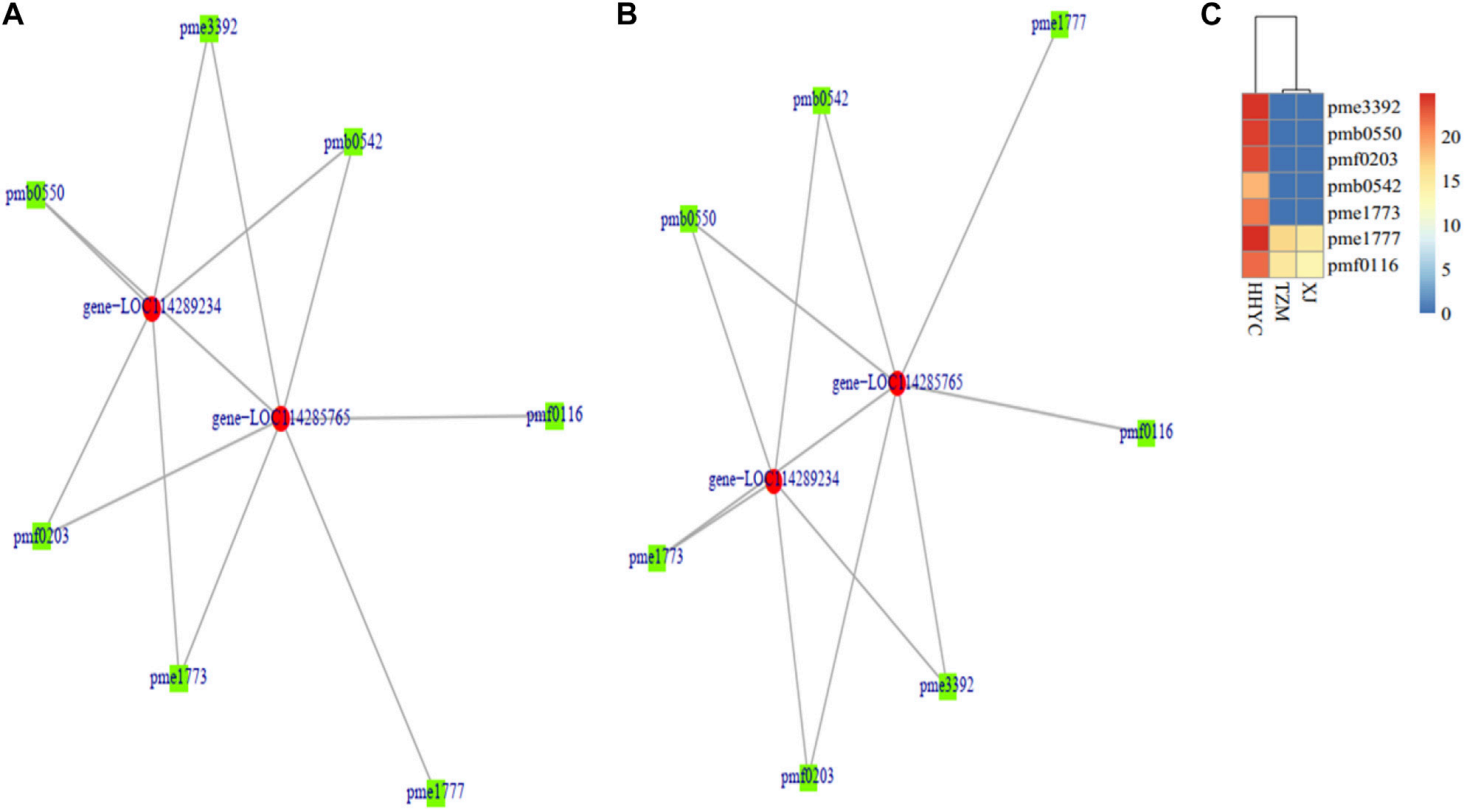
Pearson correlation network diagram of differentially expressed genes (DEGs) and differentially accumulated metabolites (DAMs) abundance in pairwise comparison of *C. reticulata* (HHYC), *C. reticulata* ‘Xuejiao’ (XJ) and *C. reticulata* ‘Tongzimian’ (TZM). **(A)**. HHYC_vs_XJ. **(B)**. HHYC_vs_TZM. **(C)**. Heatmap clustering of seven metabolites based on log2 transformed of ion intensities. The metabolites pme3392 (pelargonidin-3-O-glucoside), pmb0550 (cyanidin-3-O-glucoside (kuromanin)), pmf0203 (peonidin-3-O-glucoside), pmb0542 (cyanidin-3-O-(6″-O-malonyl) glucoside), pme1773 (cyanidin-3-O-rutinoside (keracyanin)), pme1777 (cyanidin-3,5-O-diglucoside (cyanin)) and pmf0116 (delphinidin-3,5-di-O-glucoside). The DEGs in the network (*gene-LOC114285765* and *gene-LOC114289234* encode for anthocyanidin 3-O-glucosyltransferase [EC:2.4.1.115] designated BZI in Anthocyanin biosynthetic pathway in Supplementary Figure S3). Color diagram at the right side shows the intensity of the metabolite accumulation.

A total of two DEGs (*gene-LOC114285765* and *gene-LOC114289234*) encoding for anthocyanidin 3-O-glucosyltransferase [EC:2.4.1.115] (BZI) were identified to associate (Pearson’s correlation >0.80) with seven DAMs (pme3392 (Pelargonidin-3-O-glucoside), pmb0550 (cyanidin-3-O-glucoside (Kuromanin)), pmf0203 (peonidin-3-O-glucoside), pmb0542 (cyanidin-3-O-(6″-O-malonyl)glucoside), pme1773 (cyanidin-3-O-rutinoside (keracyanin)), pme1777 (cyanidin-3,5-O-diglucoside cyanin) and pmf0116 (delphinidin-3,5-di-O-glucoside)) in both HHYC_vs_XJ and HHYC_vs_TZM (Figures 7A–C). The two genes expressed higher in HHYC than either XJ or TZM. BZI genes are responsible for the conversion of pelargonidin, cyanidin and delphinidin to pelargonidin 3-glucoside, cyanidin 3-glucoside and delphinidin 3-glucoside, respectively. Among the seven DAMs, only pme1777 (cyanidin-3,5-O-diglucoside cyanin) and pmf0116 (delphinidin-3,5-di-O-glucoside) accumulated lower in either XJ or TZM than in HHYC, while the remaining five were completely absent in both XJ and TZM (Figures 7A–C).

## 4 Discussion

Flower color is one of the most important features of horticultural crops, including *C. reticulata* (Xin et al., 2015). It does not only affect economic value, but eye appeal, aesthetics and quality. With more than 500 cultivars of *C. reticulata* with diverse flower color phenotypes (Xin et al., 2015)*,* the regulatory mechanism underlying flower coloration among the cultivars remain unknown.

Flavonoid/anthocyanins, betalains, carotenoids and chlorophylls are well known to modulate color pigmentation in plants (Mortensen, 2006; Tanaka et al., 2008; Lai et al., 2014; Zhao et al., 2017; Shen et al., 2019). With exception of chlorophylls, the other three pigmentations have been elucidated to regulate color variation depending on their synthesis, structures and subcellular localization (Zhao and Tao, 2015). Carotenoids are well documented to confer yellow, orange and red colorations (Shen et al., 2019), while anthocyanins are responsible for blue, purple, red or white colorations (Yan and Kerr, 2013). The transcriptome profile from the flowers with red, white and intermediate (pink) genotypes (HHYC, XJ and TZM, respectively) revealed that carotenoids and anthocyanins (Supplementary Table S3) co-exist which were pronounced in the red flower genotype (HHYC) (Figure 1). For instance, two ZEP genes (*gene-LOC114265684* and *novel.6342*) and one ABA-UGT (*gene-LOC114269817*) (Figure 4) involved in carotenoid biosynthesis as well as flavonoids/anthocyanins structural genes (Supplementary Table S2) including three anthocyanidin 3-O-glucosyltransferase (*gene-LOC114289234*, *gene-LOC114285765*, *gene-LOC114285774* and *gene-LOC114288492*) expressed higher in the HHYC genotype than XJ and TZM genotypes. These results highlight the co-regulation of red flower color in HHYC genotype by anthocyanins and carotenoids, with a least contribution of the latter. Our findings are consistent with the findings of Li et al. (2022); Xue et al. (2016). In addition, down-regulation/absence of anthocyanidin 3-O-glucosyltransferase is responsible for inhibition of the synthesis of delphinidin 3-glucoside (Takeda et al., 1990; Yabuya et al., 2002; Muhammad et al., 2022) and Yamazaki et al. (1999) and Nakatsuka et al. (2008) reported that a reduced level of delphinidin limits red coloration. The absence/down-regulation of anthocyanidin 3-O-glucosyltransferase genes in XJ or TZM implies that late stage of anthocyanins biosynthesis may have been blocked (Lai et al., 2013). The two ZEP genes (*gene-LOC114265684* and *novel.6342*), one ABA-UGT (*gene-LOC114269817*) and three anthocyanidin 3-O-glucosyltransferase (*gene-LOC114289234*, *gene-LOC114285765*, *gene-LOC114285774* and *gene-LOC114288492*) could be targeted to study the structural variation which is responsible for color variation between XJ and TZM. There are reports that nucleotide variation in structural genes involved in anthocyanin biosynthesis could cause variations in coloration in some plants (Ben-Simhon et al., 2015).

Transcription factors are proteins involved in converting, or transcribing DNA into RNA. It is well established the involvement of MYB-bHLH-WD40 (MBW) complex in modulating coloration in plants (Li et al., 2020a; Naik et al., 2021). Among the 61,277 expressed genes including 44,751 known genes and 16,526 novel genes were detected in the present study, no WD40 structural gene was found (Supplementary Table S1). Four MYB transcription factor genes (*gene-LOC114321352*, *gene-LOC114268549*, *gene-LOC114264400* and *gene-LOC114281024*) and four bHLH genes (*gene-LOC114233324*, *gene-LOC114258501*, *gene-LOC114298318*, *gene-LOC114287545* and *gene-LOC114319413*) expressed higher in genotypes with red color (either HHYC or TZM) than in the white pigmented genotype, XJ (Supplementary Figure S3). These suggest that MYB and bHLH transcription factors are potential positive regulators of carotenoid and anthocyanin biosynthesis in *C. reticulata*. Zhou et al. (2019) revealed that *CpbHLH1/2* modulates carotenoid biosynthesis-related genes in papaya, while He et al. (2022) demonstrated that *UpMYB44* controls carotenoid biosynthesis-related genes in *Ulva prolifera*. In comparison with other crops, MYB-bHLH role in regulating carotenoid and anthocyanin biosynthesis needs to be unearthed with genes reported in this study (Ramsay and Glover, 2005; Gonzalez et al., 2008; Zhao et al., 2012). It is important to link the identified key TFs to the target structural genes involved in the synthesis of anthocyanins through gene co-expression analysis. Such analysis will require more transcriptome data, which could be the subject of a further study.

We further performed targeted metabolome profiling with the petals of three contrasting genotypes of *C. reticulata* with the aim of identifying specific flavonoid/anthocyanin compound(s) that may account for white coloration observed in XJ and TZM genotypes relative to HHYC genotype. Targeted metabolome profiling offers absolute quantification, higher accuracy, and greater selectivity (Beckles and Roessner, 2012; Cao et al., 2020; Lelli et al., 2021). From this, 310 metabolites comprising 282 and 28 flavonoid/anthocyanin and tannin compounds, were detected, respectively (Supplementary Table S4). These compounds mostly accumulated higher in either HHYC or TZM genotype than in XJ genotype (Supplementary Table S4). For example, Luteolin-7-O-gentiobioside which is flavone compound accumulated in order of HHYC (61,965,667) > TZM (1,657,300), but was completely absent in XJ. This compound is reported to cause pink coloration in *Myoporum bontioides* (Myoporaceae) (Iwashina and Kokubugata, 2014; Cao et al., 2020). In all, eighteen anthocyanin compounds were detected among the three contrasting genotypes with wide variation in flower colors (Table 3). Of these, only 9 and 7 compounds were detected in XJ and TZM genotypes, respectively. The absence/presence of some anthocyanin compounds could be the basis for color differentiation among the three genotypes of *C. reticulata*. Cyanidin, a major anthocyanin compound which accounts for red coloration had 8 derivatives compounds (cyanidin-3-O-(6″-O-malonyl) glucoside, cyanidin-3-O-(6″-O-p-coumaroyl-2″-O-xylosyl) glucoside, cyanidin-3-O-arabinoside, cyanidin-3-O-galactoside, cyanidin-3-O-glucoside (kuromanin), and cyanidin-3-O-rutinoside (keracyanin). Of these, cyanidin-3-O-glucoside, and cyanidin-3-O-rutinoside were exclusively detected in HHYC genotype which is consistent with the findings of Kim and Kokubugata (2010) who found these compounds in red pigmented flower cultivar of buckwheat (Gan-Chao), but were absent in the white-flowered cultivar (Tanno). In another study, Li et al. (2020b) found delphinidin 3-O-rutinoside and petunidin 3-O-glucoside to be absent in white fruit of *Lycium ruthenicum* Murray. Similar observations were made among the white, violet and red flowers of *Rhododendron schlippenbachii* Maxim (Kim et al., 2013). All these indicate that the absence and reduction of anthocyanin compounds could account for the white and pink (red-white) flower coloration in XJ and TZM, respectively.

In summary, this study provides important insights into flower coloration in *C. reticulata* by unearthing key structural genes and transcription factors. This forms a foundation for biotechnological applications to explore their regulatory mechanisms in flower coloration (Kim et al., 2013; Varshney et al., 2020, 2021). Also, the flavonoid/anthocyanin compounds identified in this study could be validated and used to develop biomarkers for metabolome-assisted breeding programs (Varshney et al., 2020; Jamaloddin et al., 2021).

## Data Availability

The data presented in this study are deposited in the National Genomic Data Center under the accession number PRJCA012977 (https://ngdc.cncb.ac.cn/search/?dbId=&q=PRJCA012977).
