# Supplementary material for: Integrated transcriptome and metabolome profiling of Camellia reticulata reveal mechanisms of flower color differentiation
Source: Front Genet. 2022 Nov 22;13:1059717. doi: 10.3389/fgene.2022.1059717 (PMC9725097; doi:10.3389/fgene.2022.1059717)
Supplement: Supplementary file 3 [file Image5.pdf]

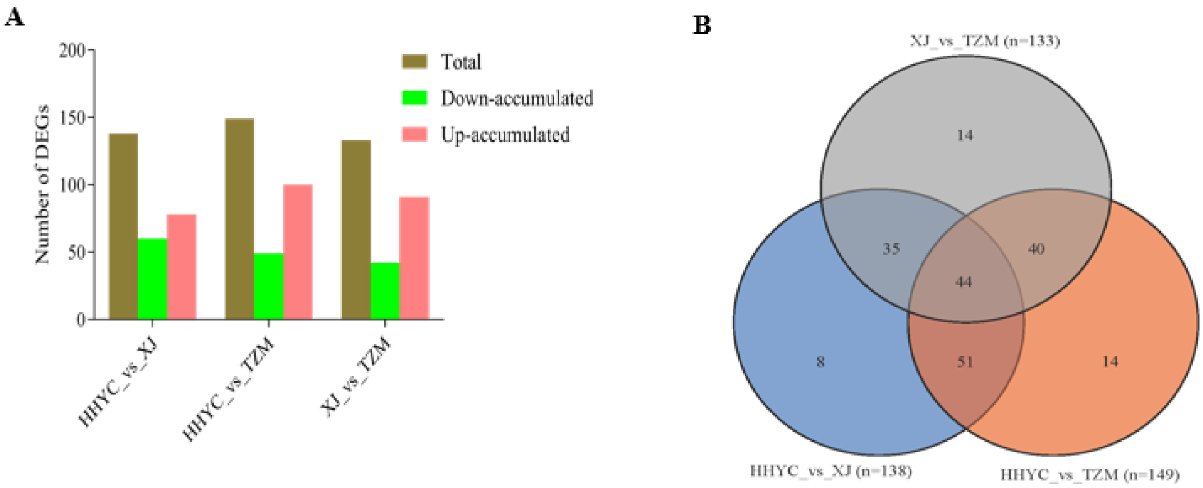

Supplementary Figure 5: Differentially accumulated metabolites (DAMs) from petals of contrasting flowers of *C. reticulata* (HHYC), *C. reticulata* 'Xuejiao' (XJ) and *C. reticulata* 'Tongzimian' (TZM). **A**. Number of DAMs and their extent of accumulation. **B**. Venn diagram of DAMs detected in the three pairwise groups (n = number of DAMs). The partial least squares discriminant analysis of log2 fold change ≥1 and variable importance in projection ≥1.
