# Supplementary material for: Integrated transcriptome and metabolome profiling of Camellia reticulata reveal mechanisms of flower color differentiation
Source: Front Genet. 2022 Nov 22;13:1059717. doi: 10.3389/fgene.2022.1059717 (PMC9725097; doi:10.3389/fgene.2022.1059717)
Supplement: Supplementary file 5 [file Image2.pdf]

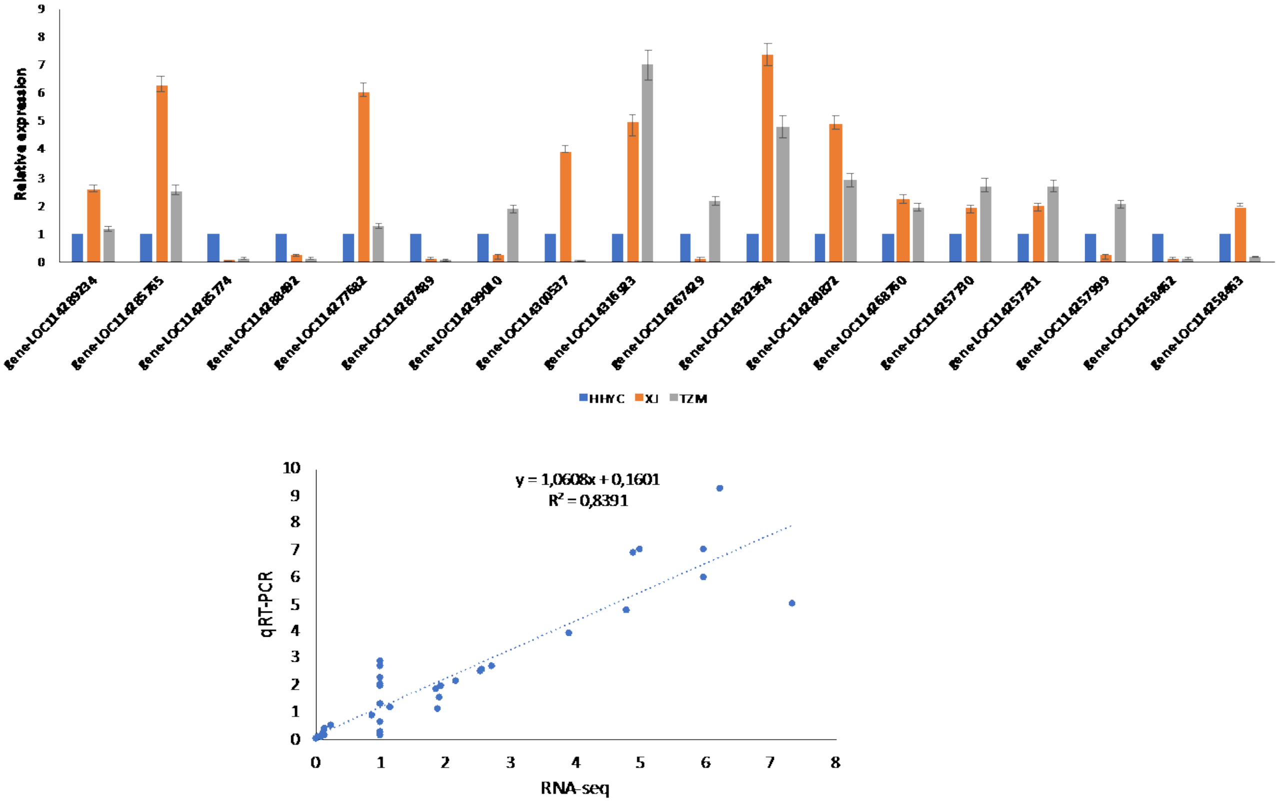

Supplementary Figure 2: qRT-PCR based verification for 18 randomly elected genes. Correlation between the RNA-seq and qRT-PCR.
