# Supplementary material for: Integrated transcriptome and metabolome profiling of Camellia reticulata reveal mechanisms of flower color differentiation
Source: Front Genet. 2022 Nov 22;13:1059717. doi: 10.3389/fgene.2022.1059717 (PMC9725097; doi:10.3389/fgene.2022.1059717)
Supplement: Supplementary file 6 [file Image3.pdf]

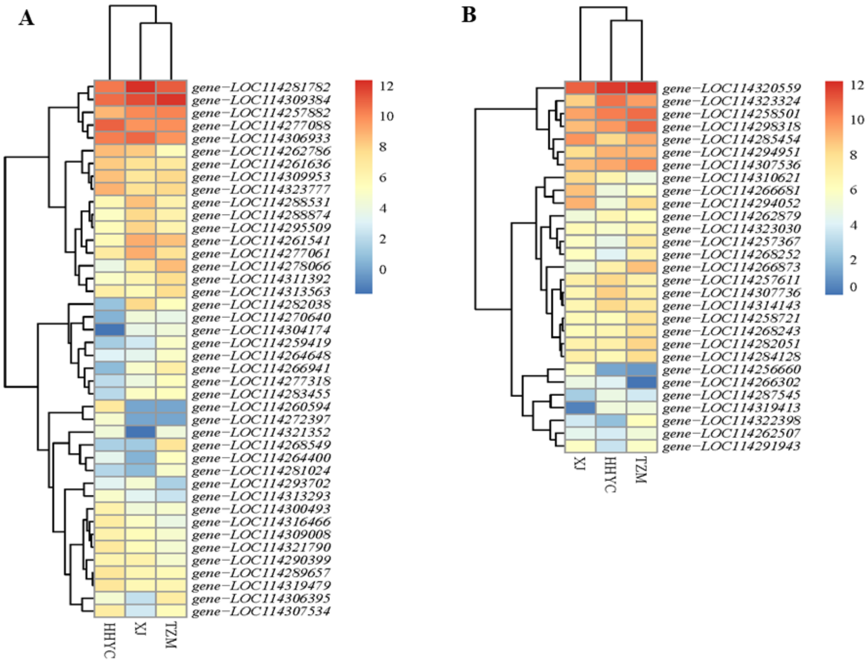

Supplementary Figure 3: Heatmap of log2 transformed of fragments per kilobase of exon per million fragments mapped of MYB-bHLH transcription factors (TF) detected among differentially expressed genes (DEGs) in the three pairwise groups of contrasting flowers of *C. reticulata* (HHYC), *C. reticulata* 'Xuejiao' (XJ) and *C. reticulata* 'Tongzimian' (TZM). **A.** MYB TF genes. **B.** bHLH TF genes. The legends for colors in each row and genotype are shown on the right-hand side of each Figure. The expression profiles were obtained as the averages of the three biological repeats.
