# Supplementary material for: Integrated transcriptome and metabolome profiling of Camellia reticulata reveal mechanisms of flower color differentiation
Source: Front Genet. 2022 Nov 22;13:1059717. doi: 10.3389/fgene.2022.1059717 (PMC9725097; doi:10.3389/fgene.2022.1059717)
Supplement: Supplementary file 10 [file Image1.pdf]

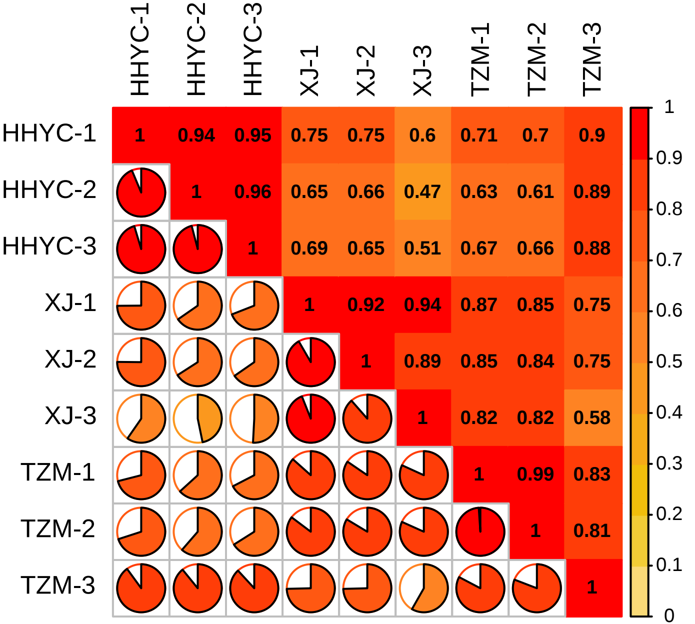

Supplementary Figure 1: Pearson correlation coefficients among the nine samples ((3 contrasting petals × 3 biological replicates) based on fragments per kilobase of exon per million fragments mapped. Three contrasting petals of *C. reticulata* (Yunnan Camellia ; HHYC1-3), *C. reticulata* 'Xuejiao' (XJ1-3) and *C. reticulata* 'Tongzimian' (TZM1-3).
