# Supplementary material for: Integrated transcriptome and metabolome profiling of Camellia reticulata reveal mechanisms of flower color differentiation
Source: Front Genet. 2022 Nov 22;13:1059717. doi: 10.3389/fgene.2022.1059717 (PMC9725097; doi:10.3389/fgene.2022.1059717)
Supplement: Supplementary file 4 [file Image4.pdf]

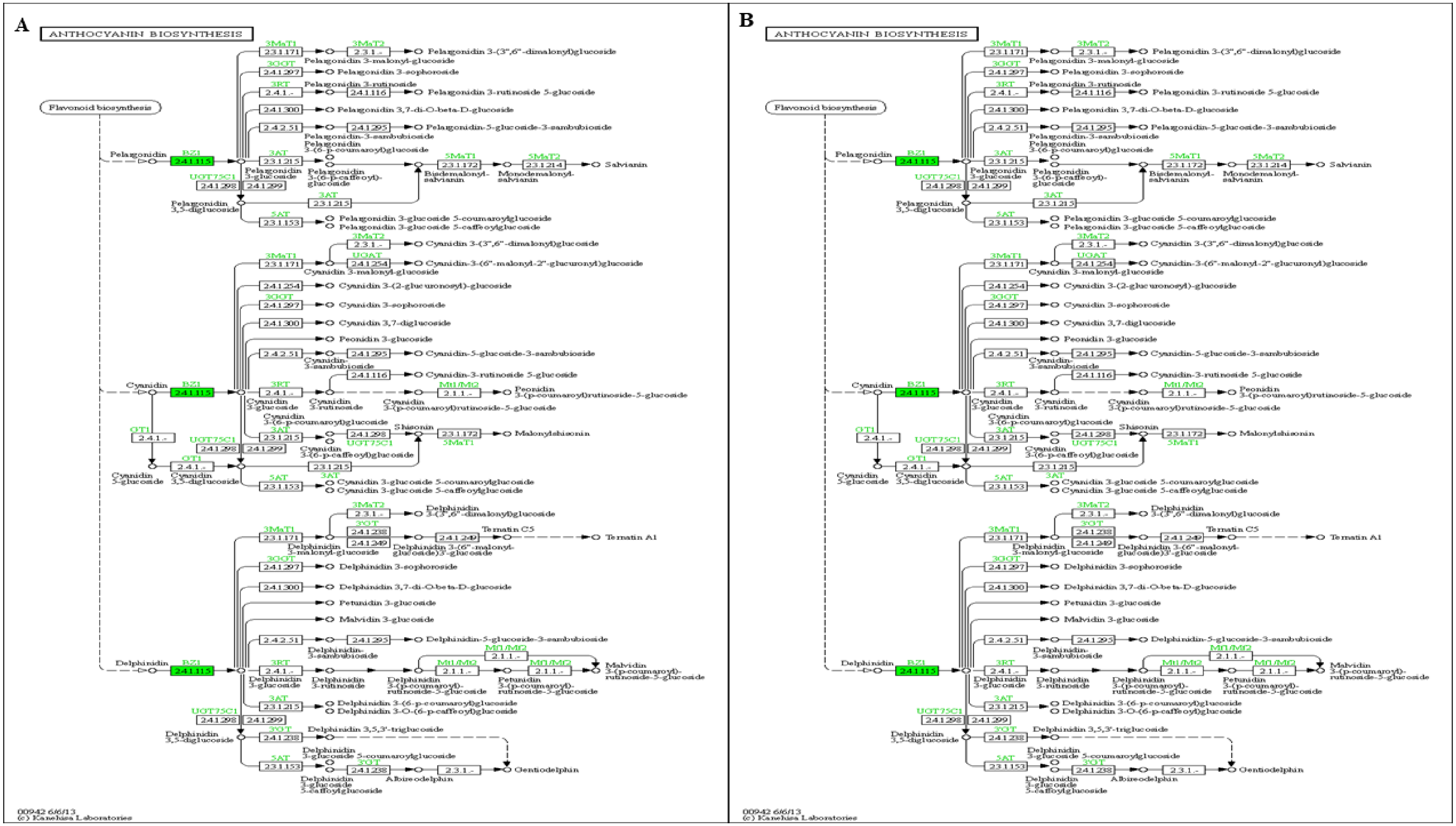

Supplementary Figure 4: Anthocyanin biosynthetic pathway in pairwise comparison of the three contrasting flowers of *Camelia reticulata* 'Yunnan Camellia' (HHYC), *C. reticulata* 'Xuejiao' (XJ) and *C. reticulata* 'Tongzimian' (TZM) retrieved from Kyoto Encyclopedia of Genes and Genomes (KEGG) pathway with differentially expressed genes (DEGs) detected in this study. **A.** HHYC_vs_XJ. **B.** HHYC_vs_TZM. The only four DEGs (*gene-LOC114289234*, *gene-LOC114285765*, *gene-LOC114285774* and *gene-LOC114288492*) encode for anthocyanidin 3-O-glucosyltransferase [EC:2.4.1.115], designated BZI. The green color of rectangle named BZI indicate the four genes expressed higher in HHYC than either XJ or TZM.
